# Thin Cord Syndrome in a Thirty-year-old Pregnant Woman

**DOI:** 10.5812/ircmj.14122

**Published:** 2014-08-05

**Authors:** Mohammadreza Hafeziahmadi, Atefeh Yousefi, Samiramis Ghavam, Sajjad Alizadeh

**Affiliations:** 1 Department of Pathology, Faculty of Medicine, Ilam University of Medical Sciences, Ilam, IR Iran; 2 Student Research Committee, Ilam University of Medical Sciences, Ilam, IR Iran; 3 Department of Cardiology, Faculty of Medicine, Ilam University of Medical Sciences, Ilam, IR Iran

**Keywords:** Umbilical Cord, Cord Syndrome, Abnormalities

## Abstract

**Introduction:**

The umbilical cord anomalies directly effect on the life quality of the fetus. It can lead to fetal death or many problems during pregnancy and delivery. Early detection of these abnormalities is of particular importance.

**Case Presentation:**

We report a case of thin cord syndrome (TCS) in a 30-year-old pregnant woman with suprapubic pain. After termination of pregnancy, histopathologic assessment confirmed the TCS.

**Conclusions:**

In all cases with suspected abortion, the umbilical cord disorder should be considered.

## 1. Introduction

The umbilical cord is a tube-shaped structure that connects the fetus to the placenta. This narrow tube-like structure delivers oxygen and nutrients to the fetus and removes the carbon dioxide and waste products of the fetus. At the five weeks after conception, the umbilical cord begins to form and continues to grow until 28 weeks of pregnancy. Typically, average length of umbilical cord is 22 to 24 inches (1). The umbilical cord contains one vein and two arteries, which are surrounded and supported by a gelatinous tissue called Wharton's jelly. The two arteries transport waste from the fetus to the placenta and the vein carries oxygen and nutrients from the placenta to the embryos (1, 2). Like other organs, a number of abnormalities can affect the umbilical cord. It may be too long, short, or even connect improperly to the placenta. Umbilical cord abnormalities can lead to fetal death or serious problems during pregnancy and delivery (3, 4). Among umbilical anomalies, Longer or shorter (< 35 cm) umbilical cords are not rare but thin cord, seen in thin cord syndrome (TCS), are very rare and are usually associated with significant pathology (5, 6). We report a very rare case of TCS that led to the fetal death.

## 2. Case Presentation

**Figure 1.**
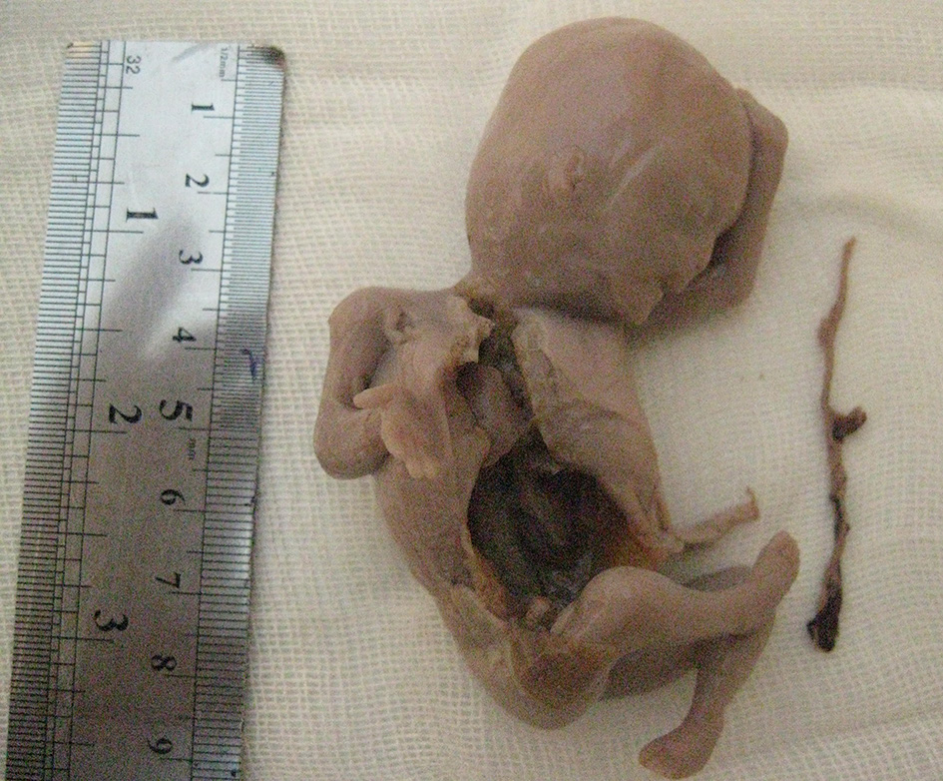
Fetal Death With Very Thin Umbilical Cord

**Figure 2.**
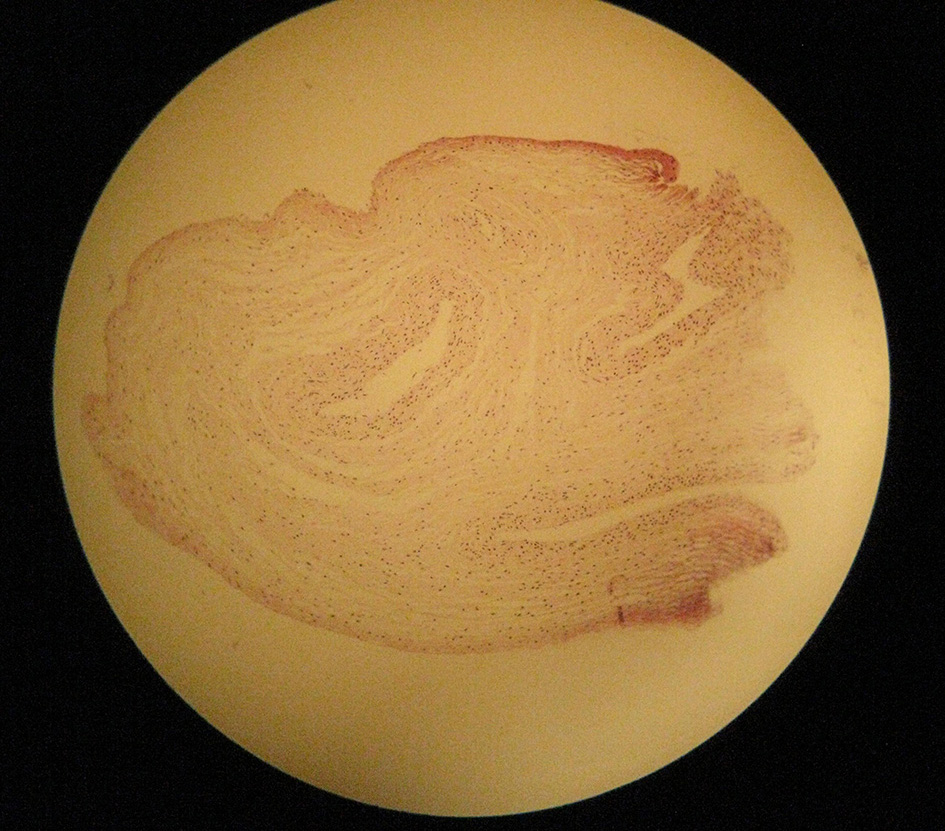
Umbilical Cord in Microscopic View

**Figure 3.**
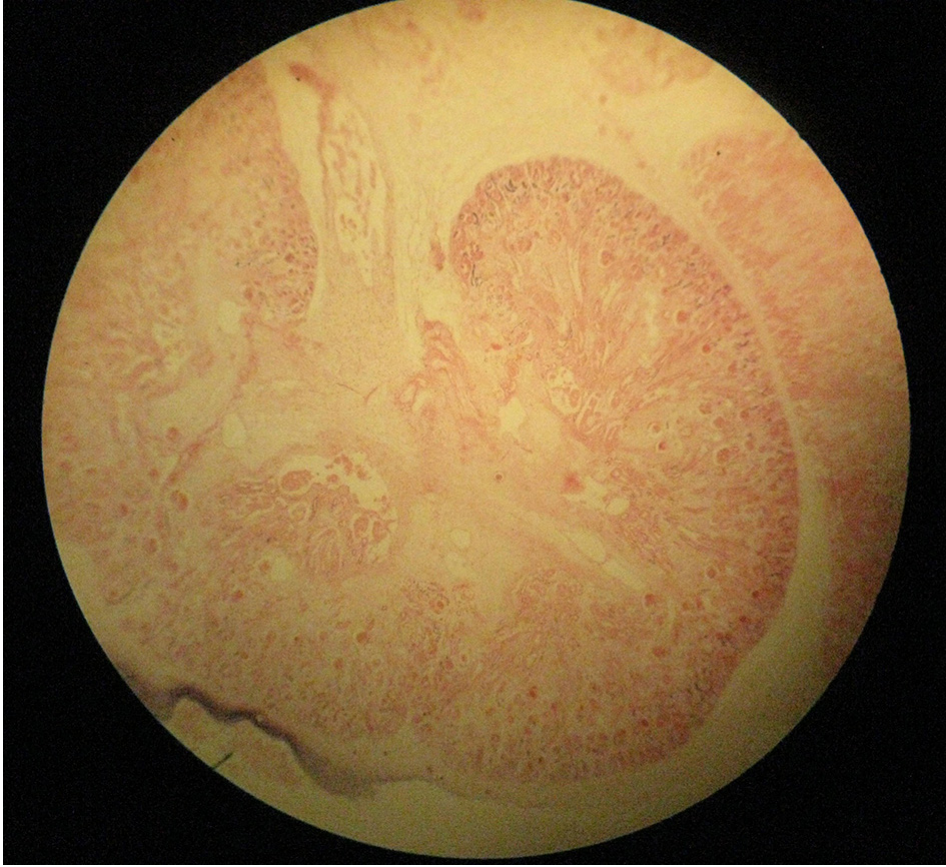
Ischemic Necrosis of Internal Organs (Kidney)

**Figure 4.**
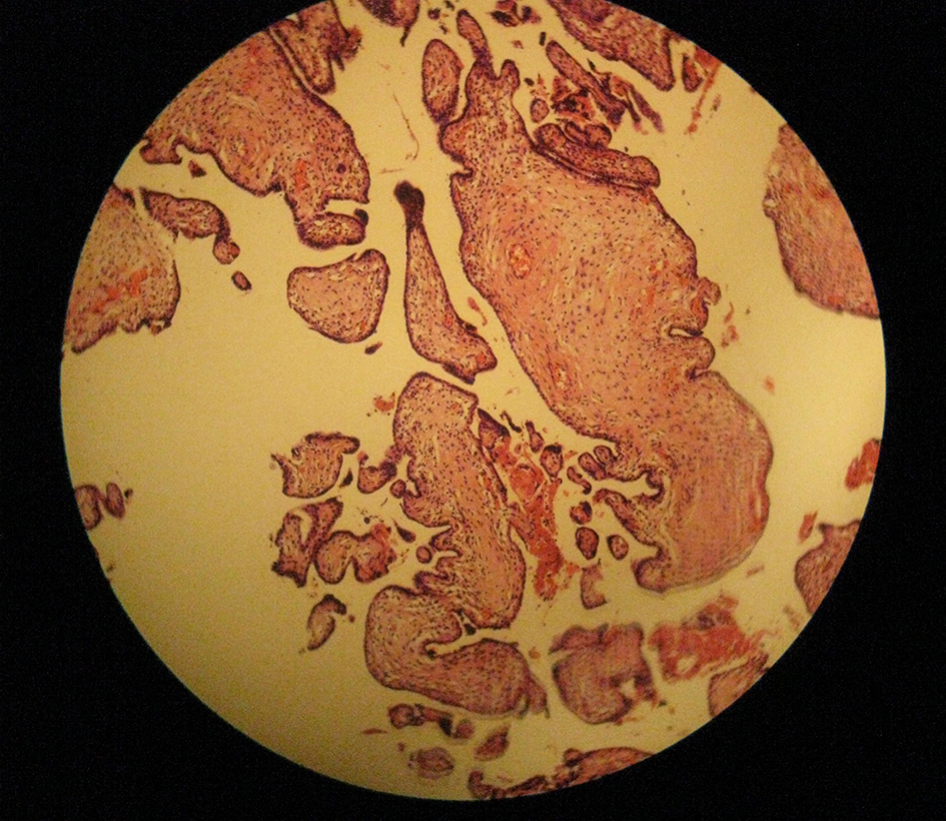
Normal Placental Tissue

A 30-year-old pregnant woman (21 weeks) from Ilam, presented with light suprapubic pain in abdomen during the previous two days. Patient had normal urinary analysis but in abdominal ultrasonography (US), her fetus had no fetal heart rate (FHR). In the next control US, no FHR was detected and the mother was referred to the gynecologist for termination of pregnancy. Our case had no history of trauma before termination. Outcome of termination of pregnancy was sent to pathology department and results showed the umbilical cord of 9 cm × 0.1 cm, which was very thin (Figures 1 and 2), impossible for detection of three vessels (in macroscopic view), and ischemic necrosis of internal organs with normal placental tissue (Figures 3 and 4). Other laboratory findings are listed in Table 1. The final diagnosis was TCS that had led to intrauterine fetal death (IUFD) with extreme and diffuse deficiency of Wharton’s jelly. At a follow-up visit, our case was asymptomatic with resolution of all signs and symptoms.

**Table 1.** Laboratory Finding^a^

	Result
**Hb, g/dL**	13.4
**FBS, mg/dL**	98
**Na, mmol/L**	139
**K, mmol/L**	4.6
**AST, U/L**	36
**ALT, U/L**	29

^a^Abbreviations: Hb, hemoglobin; FBS, fasting blood sugar; Na, sodium; K, potassium, AST, aspartate aminotransferase; and ALT, alanine aminotransferase.

## 3. Discussion

The fetus receives all of its nutrients via the umbilical cord; as a result, it is an important and vital organ for fetus. The umbilical arteries are branches of the internal iliac artery and are protected by a gelatinous stroma or Wharton’s jelly. commonly, we use the US to investigate umbilical cord and its malformations (2). It is noteworthy that from the eighth menstrual week, the umbilical cord can be visualized in US (7). Today, we can assess Wharton’s jelly of the umbilical cord by the Ultrasonic devices. These measurements are usually performed in the middle part of the umbilical cord (8). Thin cord means that circumference of umbilical cord is less than 1 cm and is associated with postdates or small for gestational age births. The normal diameter of the umbilical cord in a normal term infant is 1.5 cm × 36 cm (9, 10). TCS is a rare anomaly that is defined with short and thin umbilical cord. It frequently leads to IUFD and abortion. Deficiency of Wharton’s jelly is common among fetuses with TCS; hence, compression of vessels is a more possible than when they are protected. TCS occur more often with growth-retarded fetuses and in preeclampsia, but idiopathic causes also exist. In conclusion, in all cases of suspected abortion, the umbilical cord disease should be considered in the differential diagnosis.
